# CO_2_ ‐induced biochemical changes in leaf volatiles decreased fire‐intensity in the run‐up to the Triassic–Jurassic boundary

**DOI:** 10.1111/nph.18299

**Published:** 2022-06-30

**Authors:** Sarah J. Baker, Rebecca A. Dewhirst, Jennifer C. McElwain, Matthew Haworth, Claire M. Belcher

**Affiliations:** ^1^ wildFIRE Lab University of Exeter Exeter EX4 4PS UK; ^2^ Botany Department, School of Natural Sciences Trinity College Dublin Dublin D02 PN40 Ireland; ^3^ Institute for Sustainable Plant Protection National Research Council (CNR‐IPSP) Via Madonna del Piano 10 Sesto Fiorentino Florence Firenze 50019 Italy

**Keywords:** CO_2_‐induced biochemical changes, fire behaviour, fire intensity, leaf volatiles, lignin content, Triassic–Jurassic transition

## Abstract

- The Triassic–Jurassic boundary marks the third largest mass extinction event in the Phanerozoic, characterized by a rise in CO_2_‐concentrations from *c.* 600 ppm to *c.* 2100–2400 ppm, coupled with a *c.* 3.0–4.0°C temperature rise. This is hypothesized to have induced major floral turnover, altering vegetation structure, composition and leaf morphology, which in turn are hypothesized to have driven changes in wildfire. However, the effects of elevated CO_2_ on fuel properties, such as chemical composition of leaves, are also important in influencing fire behaviour, but yet have not been considered.
- We test this by selecting three Triassic analogue species grown experimentally in different atmospheric compositions, and analyse variations in leaf chemistry, and leaf level flammability. These data were used to inform a fire behaviour model.
- We find that all three species tested showed a reduction in their volatile component, leading to lower flammability. Accounting for these variations in a model, our results suggest that leaf intrinsic flammability has a measurable impact on modelled fire behaviour.
- If scaled up to ecosystem level, periods of elevated CO_2_ may therefore be capable of inducing both biochemical and morphological changes in fuel properties, and thus may be capable of influencing fire behaviour.

## Introduction

The Triassic–Jurassic boundary transition (TJT) marks one of the largest mass extinction events in the Phanerozoic (Fowell & Olsen, 1993; McElwain *et al*., 1999; Ward *et al*., 2001). At Astartekløft, East Greenland, stomatal *p*CO_2_ proxy analysis of macrofossil leaves preserved, suggest CO_2_ concentrations rose from *c*. 600 ppm to *c*. 2100–2400 ppm, where CO_2_ concentrations rose gradually during the Rhaetian, before doubling at/slightly after the boundary (McElwain *et al*., 1999; Steinthorsdottir *et al*., 2011), driving a *c*. 3.0–4.0°C temperature rise across the TJT (McElwain *et al*., 1999). In East Greenland, modelled local summer temperatures are estimated to have risen to 36°C (Huynh & Poulsen, 2005; Sellwood & Valdes, 2006).

These CO_2_‐climate driven changes are hypothesized to have induced major floral turnover, altering vegetation structure, composition and leaf morphology (e.g. McElwain *et al*., 2007; Belcher *et al*., 2010). This, coupled with an increased probability of ignition through increased lightning strikes under higher atmospheric CO_2_ conditions are hypothesized to have driven changes in wildfire activity and behaviour across the TJT (Belcher *et al*., 2010; Peterson & Linström, 2012; Belcher, 2016). Here, evidence of wildfire in the form of fossil charcoal and polycyclic aromatic hydrocarbons (PAHs) have been well documented from numerous locations (e.g. Harris, 1957, 1958; Marynowski & Simoneit, 2009; Peterson & Linström, 2012; Song *et al*., 2020).

However, records of fossil charcoal and other combustion residues are difficult to interpret in deep time because these signatures rarely represent individual fire events, but rather overall rises in fire activity during long periods of time. Moreover, fires themselves create varied abundances of these products depending on the nature of combustion. Recent research has indicated for example, that the abundance of charcoal produced by fires varies according to plant species, which is related to the intrinsic physical and chemical properties of the fuel that leads to different fire behaviours (Hudspith *et al*., 2017). This means that not only will different fires produce different amounts and sizes of char (Crawford & Belcher, 2016; Hudspith *et al*., 2017) but that also certain taxa will be over represented in fossil charcoal assemblages and some indeed not preserved at all (Hudspith *et al*., 2017; Hudspith & Belcher, 2017).

Perhaps more importantly fire frequency is only one aspect of fire regime that determines the effects of fire on vegetation shifts and its ecological impact. More significant is that of the fire behaviour and fire severity, hence estimates of palaeofire behaviour are required for the ancient past if we are to understand the impacts of significant shifts in fire regime on ecosystems. A shift in fire regime has previously been suggested across the TJT at Astartekløft, where prior to the rise in CO_2_ ecosystems are hypothesized to have experienced infrequent but intense fires. Then as CO_2_ concentrations rose fires are hypothesized to have become more frequent but of lower intensity (Belcher *et al*., 2010; Belcher, 2016). These changes were inferred by coupling variations in the abundance of fossil charcoal with flammability metrics derived from laboratory experiments linked to changes in the leaf morphology of dominant species across the TJT. Here we expand this approach by modelling fire behaviour across the TJT by constructing fuel models based on the shifts in plant dominance observed at Astartekløft (McElwain *et al*., 2007; Belcher *et al*., 2010). We utilize the model behaveplus (Andrews, 2010) which requires the heat content of the fuel components. To gather this information we have undertaken unique plant growth experiments to explore the effect of changing atmosphere CO_2_ on the volatile components of plants, which are known to be important in influencing fire behaviour (Belcher, 2013; Hudspith *et al*., 2017; Dewhirst *et al*., 2020). For example, an astounding array of secondary metabolites including thousands of volatile compounds play a range of roles including herbivory defence, signalling, and immunity in plants. Some of these compounds, such as terpenoids (specifically monoterpenes and sesquiterpenes) also contribute to leaf flammability due to their low flash points (e.g. Owens *et al*., 1998; Pausas *et al*., 2016; Dewhirst *et al*., 2020).

Changes in temperature, atmospheric CO_2_ levels, aridity, and drought, can influence changes in leaf morphology and chemistry, as well as bark traits which in turn affect fire behaviour (Popović *et al*., 2021; Scalon *et al*., 2021). Warming leads to a decline in photosynthetic rate, as Rubisco favours oxygenation rather than carboxylation at higher temperatures (Berry & Björkman, 1980), leading to lower carbon fixation (Haworth *et al*., 2018). Increased CO_2_ however provides more substrate for photosynthesis and so increases carbon assimilation (Berry & Björkman, 1980). As such plants grown under elevated CO_2_ have been hypothesized to accumulate greater levels of carbon‐based secondary metabolites (Peñuelas *et al*., 1997). This is further supported by an observed increase in community weighted leaf mass per area across the TJT, which based on leaf economic spectrum (Wright *et al*., 2004) supports greater levels of carbon‐based secondary metabolites in the earliest Jurassic and TJT leaves (Soh *et al*., 2017). However, the observed response to elevated CO_2_ in various experimental trials has been varied.

Warming and elevated CO_2_ have been reported to have contrasting effects on secondary metabolites, including terpenes (Valor *et al*., 2017); and reviewed in Holopainen *et al*. (2018) and Feng *et al*. (2019). In gymnosperms, foliar terpenes increased with warming (Zvereva & Kozlov, 2005), whereas they decreased with elevated CO_2_ in *Pinus sylvestris* and *Picea abies* (Sallas *et al*., 2003). Monoterpene production was downregulated under elevated CO_2_ resulting in a decrease in monoterpenes in terpene‐storing conifers (De Lillis *et al*., 2009).

Different compound types respond in different ways to elevated CO_2_; for instance, phenolic compounds tend to increase under elevated CO_2_ (e.g. Peltonen *et al*., 2005) whereas terpenoid compounds tend to decrease (Holopainen *et al*., 2018). The opposite is true for elevated temperature, under which phenolics decreased and terpenes increased. Under elevated CO_2_ phenolic compounds in *Salix myrsinifolia* were found to increase, but decrease under elevated temperatures. The combination of elevated CO_2_ and temperature resulted in no change in total shoot phenolics, but a decrease in the total phenolic concentration (Veteli *et al*., 2002).

Meta‐analysis revealed that ‘leaf toughness’ increased under elevated CO_2_ (Zvereva & Kozlov, 2005; Stiling & Cornelissen, 2007; Robinson *et al*., 2012). Leaf toughness is potentially linked to lignin, as lignin is an important structural component of plants. Lignin is one of the most recalcitrant plant compounds and lignin‐rich leaf litter has a slower rate of decomposition than lignin‐poor litter (Wedderburn & Carter, 1999). This has an impact on the accumulation of fuels required for surface fires; high‐lignin litter will accumulate to greater amounts than easily decomposed low‐lignin litter and so increase the probability of a surface fire occurring.

Hence, we hypothesize that CO_2_‐driven climate changes in the run‐up to the TJT may have been capable of not only inducing changes in leaf morphological fuel properties (Belcher *et al*., 2010; Belcher, 2016), but also variations in biochemical properties, which together alter wildfire behaviour (Dewhirst *et al*., 2020). We selected three species with ancient evolutionary origins (the gymnosperms *Agathis australis*, *Ginkgo biloba* and the fern *Dicksonia antartica*) that correspond to morphotypes of the dominant litter‐forming vegetation observed at the Astartekløft site (McElwain *et al*., 2007; Belcher, 2016). We grew these species in current ambient and elevated CO_2_ (Triassic–Jurassic) atmospheric conditions for 18 months and analysed variations in the chemistry of the leaves (terpene and lignin content), using gas chromatography–mass spectrometry (GC–MS), and aspects of their leaf level flammability using micro‐calorimetry. These data were used to inform a fire behaviour model (behaveplus; Andrews, 2010) to produce estimates in variations in surface fire rate of spread, fireline intensity and canopy scorch height based on the increase in [CO_2_] and global temperatures in the run‐up to the TJT and the floral changes observed in plant macrofossils at Astartekløft, East Greenland (McElwain *et al*., 2007; Belcher, 2016).

## Materials and Methods

### Study site

The site at Astartekløft comprises eight sedimentary rock horizons, termed ‘plant beds’, that span the TJT and contain an abundance of well‐preserved macrofossils (Harris, 1935; McElwain *et al*., 2007) (> 3000 census collected fossil leaves), thus enabling the reconstruction of surface litter and surface fuel for fire reconstructions (Belcher, 2016). Here, the Triassic comprises plant beds 1, 1.5, 2, 3, 4, and 5 where plant bed 5 is split into 5A and 5B that represents the shift in leaf morphology, during the Triassic–Jurassic transition (Belcher *et al*., 2010). The increase in charcoal abundance has been observed in plant bed 5B.

### Plant growth experiments

#### Volatile content and flammability

Plants were grown in Conviron BDW‐40 walk‐in growth chambers at University College Dublin, Ireland, at ambient (380 μmol mol^−1^) and elevated (1500 μmol mol^−1^) [CO_2_]. All other growth conditions remained constant between [CO_2_] treatments: 600 μmol m^−2^ s^−1^ photosynthetically active radiation for 16 h d^−1^ with 1 h simulated dawn : dusk, day : night time temperature regime of 28°C : 18°C, relative humidity of 80% and irrigation with 60 ml of water per day. Leaves were fully expanded and from the top of the canopy receiving full illumination. Only leaves that had formed during the chambers after 18 months were used, and were collected at the same time to eliminate reactions to herbivory. Leaves were then placed in paper envelopes and allowed to dry.

#### Volatile compound analysis

Volatiles were extracted from dried leaves (two leaves per plant from two plants, see Supporting Information Tables S1, S2) ground in glass pestle and mortar under liquid nitrogen in *n*‐hexane (0.01 g (dry weight) in 1 ml hexane) with 10 μM butylated hydroxytoluene (BHT; as an internal standard) by sonication and subsequent overnight incubation. Samples were analysed using an Agilent 7200 series accurate mass quadrupole time‐of‐flight (Q‐TOF) mass spectrometer coupled to a 7890A GC system (Agilent Technologies, Santa Clara, CA, USA), equipped with an EI (electron ionization) ion source. Briefly, 5 μl of each sample was injected into a nondeactivated, baffled glass liner with a 12 : 1 split ratio (14.448 ml min^−1^ split flow) and the inlet temperature was maintained at 250°C. A Zebron semi‐volatiles (Phenomenex, Torrance, CA, USA) column (30 m × 250 μm × 0.25 μm) coupled with a 10 m guard column, was maintained at a constant helium flow of 1.2 ml min^−1^. The temperature of the column was ramped up at a rate of 15°C min^−1^, from 70°C to 310°C over 16 min, and then held at 310°C for a further 6 min. The EI source emission current and voltage were held at 35 μA and 70 eV, respectively. The mass range was set from 50 to 600 *m*/*z*, with an acquisition rate of 5 spectra s^−1^, and a solvent delay of 4 min. Data were analysed using Agilent MassHunter Qualitative Analysis software (v.B.07.00) and compounds identified where possible by comparison with standards and National Institute of Standards and Technology (NIST 11 Mass Spectral Library) and Golm libraries (Hummel *et al*., 2007). See Figs S1–S3 for replicate GC profiles.

#### Lignin analysis

Protein‐free cell wall preparations were obtained for two leaves per plant from two plants by sequential washing in pH 7 potassium phosphate buffer (0.1 M), 1 M sodium chloride (NaCl) solution, 1% (v/v) Triton‐X100 and acetone. Acid‐soluble lignin was assayed using the acetyl bromide method (Moreira‐Vilar *et al*., 2014), and the absorbance measured at 280 nm.

#### Leaf‐level flammability analysis

The intrinsic flammability of the leaves was measured using a Federal Aviation Administration (FAA) microcalorimeter (Fire Testing Technology, East Grinstead, UK) that was developed to allow direct measurements of heat release rate in respect to material properties and chemical composition of materials. The FAA microcalorimeter is a pyrolysis combustion flow calorimeter and was used to reproduce the solid‐state and gas phase processes of flaming combustion by heating 10–15 mg samples of each leaf type in an inert gas stream (nitrogen gas) where the volatile gases are driven off and oxidized at high‐temperature in excess oxygen. The microcalorimeter then measures the rate of heat release based on the oxygen consumption history of the fuel. The samples were exposed to a heating programme that ramped up to 750°C at a rate of 3°C s^−1^. Two leaves per plant from two plants were analysed each in duplicate. The peak heat release rate (pHRR: the most intense flux of heat during the combustion of the leaf material, indicates the maximum decomposition rate of the leaves which is related to the volatile gas flux of the material), heat capacity (HRC: the maximum capability of the leaf material to release combustion heat per degree of temperature during pyrolysis; this measure provides an indication of the resistance of the leaves to thermal degradation) and total heat release (THR: the total energy released by the leaf during combustion) was determined for each leaf on a per gram dry mass basis. The THR was used to inform the heat content aspect of the fuel models run in behaveplus for each fuel type.

#### Estimating changes in fire behaviour in the run‐up to the TJT

We utilized the behaveplus modelling system (Andrews, 2010) to estimate alterations in fire behaviour across the TJT. behaveplus is used in predicting fire behaviour in modern US ecosystems and has also been used to estimate changes in Cretaceous fire behaviour (Belcher & Hudspith, 2017). behaveplus consists of a set of mathematical models and requires simple input parameters that detail fuel characteristics and environmental conditions, such as dead and live fuel loads, fuel bed depth, moisture of extinction and live heat contents (see Notes S1 for full details). Here, calculations are included for surface fire rate of spread, fireline intensity and flame length, reaction intensity and heat per unit area, intermediate values of heat source, heat sink, dead fuel moisture and many more (see Andrews, 2014). We modelled the behaviour of surface fires and their ability to scorch the canopy. We did not predict crown fire behaviour because the information that is required (for example, surface fuel moisture and canopy base height) is currently beyond the state‐of‐the‐art for palaeontological observations. Hence, we have modelled interactions between dead litter fuel, surface level fuels (such as ferns) and the reach of the effects of the surface fires on the canopy.

A set of fuel models that numerically describe the Triassic and transitional surface fuels based on the major canopy‐ and litter‐forming morphotypes, coupled with the change in vegetation documented within each plant bed, were constructed (McElwain *et al*., 2007; Belcher, 2016). The descriptions were made using details of the litter forming fuel types present, as outlined in the genus‐level relative abundance of the plant fossils found at Astartekloft used in Belcher *et al*. (2010) (see their SI 41561_2010_BFngeo871_MOESM293_ESM.xls)), and their morphology (Belcher, 2016). The relative percentages for each ecosystem element’s influence on each fuel model is indicated in Table S3 and the numeric descriptors of all the fuel models used are shown in Table S4.

#### Vegetation Phase 1 (plant beds 1, 1.5 and 2 Triassic age)

In these plant beds the canopy and main litter forming species consisted of broad‐leaved fuels of Ginkgos and the conifer *Podozamites*, whilst the sub‐canopy likely consisted of ferns, cycads and bennettites (with ferns as the dominant) (McElwain *et al*., 2007; Belcher *et al*., 2010; Belcher, 2016) (Table S3). During Vegetation Phase 2 (plant beds 3, 4 and 5A Triassic to latest Triassic age), the sub‐canopy habitat is diminished, with the loss of cycads and bennettites. Ginkgos are also lost and osmundaceous ferns replace dipterid ferns (McElwain *et al*., 2007; Belcher, 2016). During this phase, interpreted litter fuels remain dominated by broad‐leaved conifer leaf‐shed litter of *Podozamites* (Belcher, 2016) (Table S3). The fuel models for all plant beds 1 through to 5A are based on a Timber‐Understory fuel model – TU5 of Scott & Burgan (2005) (Table S4). TU5 is a timber understory fuel model and representative of broad‐leaved fuel, to represent the broad‐leaved surface litter likely, from a canopy of Ginkgo and *Podozamites* (making up the dead litter fuel class), coupled with the sub‐canopy of ferns, cycads and bennettites (dominating the live surface fuel load).

#### Vegetation Phase 3

In plant bed 5B, narrow‐leaved shoot‐shedding conifers are hypothesized to become dominant (Stachyotaxus), and abundances of ferns, cycads and bennettites become limited (McElwain *et al*., 2007; Belcher *et al*., 2010; Belcher, 2016). To account for this switch in inferred dominance from broad‐leaved to narrow‐leaved litter species, a different fuel model is used which is based on a Timber‐Litter fuel model – TL8 of Scott & Burgan (2005) (Table S4). TL8 represents a forest ecosystem with little understory but that maintains the presence of needle leaf litter, capturing the shift to a narrow leaf morphotype.

To account for the interpreted changing vegetation composition and dominant litter‐forming morphotype changes, the fuel models were adjusted for each plant bed. Many elements in the chosen fuel models were kept as standard throughout and are described in the Notes S1 and in Table S4. Here we describe the changes that were made between the fuel models within each vegetation phase.

Because our plant growth experiments indicated differences in heat content between species and a clear decrease in the heat content (THR) between leaves grown under control and high CO_2_ conditions (Table 1), we altered the heat content in each fuel model according to shifts in plant dominance and environmental changes. The heat content for each of the plant beds fuel model were informed using values from modern day morphotypes or nearest living relatives from published data taken from Belcher (2016) and Belcher & Hudspith (2017). The dead fuel (litter) heat content for each palaeo‐fuel model was then established by weighting according to the dominance of the major litter‐forming fossil genera represented in each plant bed from Belcher (2016) and by adjusting this according to the proportion of change we observed between control and high CO_2_ (see Table S3). As an example, in plant bed 1, *Ginkgo* makes up 38% of the dominant litter morphotype, and *Podozamites* makes up 62% (Belcher *et al*., 2010; Belcher, 2016), hence a weighting of 62% was assigned to the dead fuel heat content of *Agathis* (a nearest living morphotype) and 38% weighted from the heat content of *Ginkgo* (the nearest living relative) (see Table S3). This approach was used for all plant beds (see Tables S3, S4).

**Table 1:** Summary of volatile compound peaks from gas chromatography–mass spectrometry (GC–MS) for each species.

Compound class	*Dicksonia*	*Ginkgo*	*Agathis*
Monoterpene	—	—	40, 41, 42, 43, 44
Sesquiterpene	—	18	45, 46, 47, 48, 49
Diterpene	2, 3	3, 19, 22, 23, 25	2, 3, 19, 22, 23, 25, 50, 51
Fatty acid	—	13, 14, 20, 21, 26	13, 20
Fatty alcohol	—	12, 38	38
Long‐chain hydrocarbon	5, 6, 7, 8, 9, 10	15, 16, 29, 35, 36	15, 29, 35, 36, 53, 54
Phenolic	—	28	—
Isoprenoid ketone	1	1	1
Unknown	4	11, 17, 24, 27, 30, 31, 32, 33, 34, 37, 39	17, 54

For the TJT beds 5A and 5B, heat content was further adjusted accordingly for the effects of elevated [CO_2_] based on the plant biochemistry and the resultant flammability using the findings of our plant growth experiments. For example, *A. australis* saw a 35% reduction in dead fuel heat content under elevated [CO_2_] (Tables 1, S3, S4). For the TJT beds, a range of scenarios were run to allow us to consider how changes in; (a) fuel alone, (b) fuel and increasing [CO_2_], (c) fuel, increasing [CO_2_] plus and an assumed 4°C rise in global temperature (McElwain *et al*., 1999) and (d) fuel, [CO_2_] plus and an assumed 11°C rise in regional summertime peak temperature (Huynh & Poulsen, 2005) would alter the fire behaviour (see Table 2). All live fuel heat contents, i.e. that of the understory/sub‐canopy were derived from *D. antarctica* as a nearest living morphotype where the data was taken from Belcher & Hudspith (2017) and adjusted as noted earlier.

**Table 2:** Measures of flammability and percentage change between high and ambient CO_2_.

Vegetation type	Mean total heat release control conditions (KJ g^−1^)	Mean total heat release under elevated CO_2_ (KJ g^−1^)	Percentage change in mean total heat release between control and elevated CO_2_ (%)	Mean heat release capacity control conditions (J g^−1^ K^−1^)	Mean heat release capacity under elevated CO_2_ (J g^−1^ K^−1^)	Percentage change in mean heat release capacity between control and elevated CO_2_ (%)
*Dicksonia*	7.65 (±0.15)	7.35 (±0.17)	4	58 (±2.26)	50.5 (±0.58)	13
*Ginkgo*	9.5 (±0.25)	7.38 (±0.34)	22	58 (±4.69)	42.75 (±2.92)	26
*Agathis*	11.37 (±0.24)	7.35 (±0.03)	35	84.75 (±1.66)	45.75 (±0.55)	46

*n* = 4 in each case, two leaves per plant from two plants were analysed. The standard error for each measurement is indicated in parentheses.

Two further changes were made. (1) To represent the loss in sub‐canopy of cycads and bennettites in Vegetation Phase 2 (plant beds 3, 4 and 5A), the live herbaceous fuel load was halved and further adjustments in this fuel category were made for bed 5B (see Table S4 for information on changes to the live herbaceous fuel load). (2) To represent the debate surrounding changes in leaf litter fuel load across the TJT, we ran two options for beds 5A and 5B. The first assumed that a 25% decrease in lignin content would lead to a lower load of 1 h fuel and a second with a high fuel based on the increase in leaf mass per area across the TJT (Soh *et al*., 2017). Hence, we ran one set of options with a lower 1 h fuel load and another set assuming the standard fuel load of the TL1 fuel.

Each fuel model created was added as a custom fuel model into the behaveplus fire behaviour modelling program (Andrews, 2010). We modelled outputs of surface spread rate, surface fireline intensity and scorch height for fires carried in the understory fuels only. Surface spread rate refers to the speed at which a fire travels through the surface fuels. Fireline intensity refers to the amount of energy released per unit of time per unit length of the fire front, whilst scorch height is the height at which the surface fire will begin to dry and scorch canopy fuels. Higher output values for each of these parameters allows for better pre‐heating and drying of fuels ahead of a fire, thus enabling the fire to propagate more easily. For example, increased scorch height and fireline intensity (which also relates to flame length) allow for heating and scorching of canopy fuels, increasing the probability that a surface fire will transition into a crown fire. The parameters required to produce these estimates are: Fuel model (described in Tables S2, S3), dead surface fuel moisture (set at 8% in all runs), live surface fuel moisture (set at 100% in all runs), slope angle (set at 30% for all runs) and temperature. These choices are explained in Notes S1. We ran two alternatives for mid‐flame wind speed, of 5.6 km h^−1^ and 11.2 km h^−1^, where mid‐flame windspeed describes the wind speed at eye height, beneath a forest canopy. We selected relatively low mid‐flame wind speeds, because the vegetation at Astartekloft is believed to be considered to have been a relatively dense subtropical forest (McElwain *et al*., 2007).

## Results

### Changes in leaf biochemistry

**Fig. 1:**
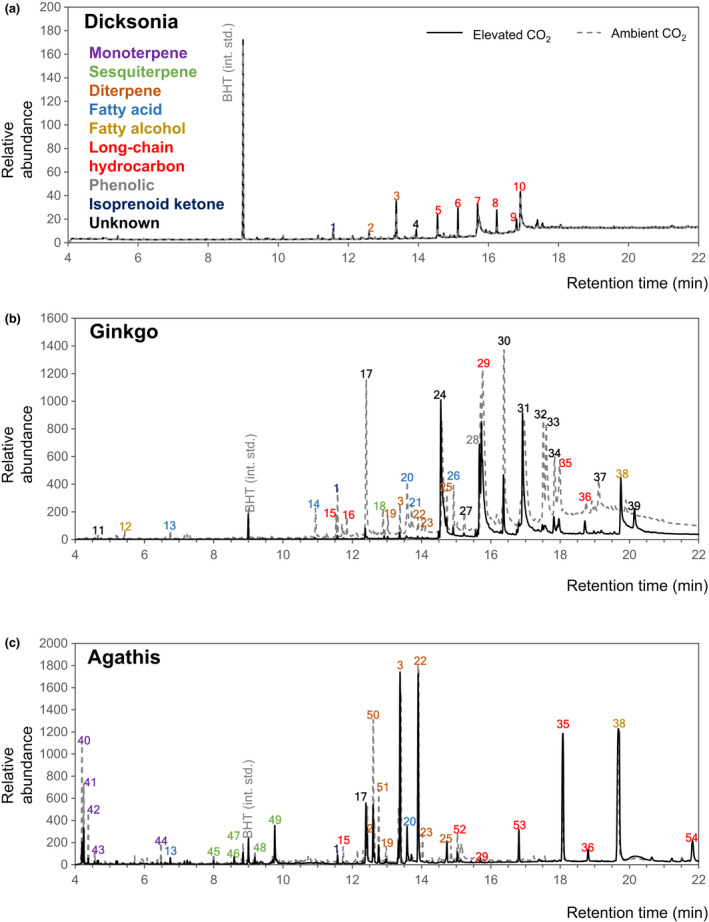
Volatile compound representative profiles of *Dicksonia*, *Ginkgo* and *Agathis*. Volatile compounds were extracted from dried samples of *Dicksonia* (a), *Ginkgo* (b) and *Agathis* (c) in hexane with an internal standard of butylated hydroxytoluene, and analysed by gas chromatography–mass spectrometry. Peaks representing individual compounds are numbered. Where possible peaks were identified to compounds class level, as in Table 1. More detailed information for individual compounds can be found in Supporting Information Figs S1–S3. Representative profiles selected out of three replicates.

Representative GC−MS profiles for each species are shown in Fig. 1 with each producing distinct volatile compounds. *Dicksonia antarctica* had the lowest abundance of compounds, and *A. australis* had the highest (Fig. 1). *Dicksonia antarctica* predominantly contains long‐chain alkane hydrocarbons whilst, *A. australis* is the only species to contain monoterpenes. *Ginkgo biloba* and *A. australis* both contain sesquiterpenes, to varying degrees, and all three species contain at least two diterpenes (Table 1). There were two compounds common to all species; an isoprenoid ketone (trimethyl pentadecanone) and a diterpene (kaur‐16‐ene). In addition to kaur‐16‐ene, *D. antarctica* predominantly contained long‐chain alkane hydrocarbons, whilst *A. australis* extracts contained a significant proportion of terpenes, including monoterpenes, sesquiterpenes and diterpenes, along with some long‐chain hydrocarbons. *Ginkgo biloba* contained several isoprenoid‐related compounds, including several diterpenes and a sesquiterpene.

**Fig. 2:**
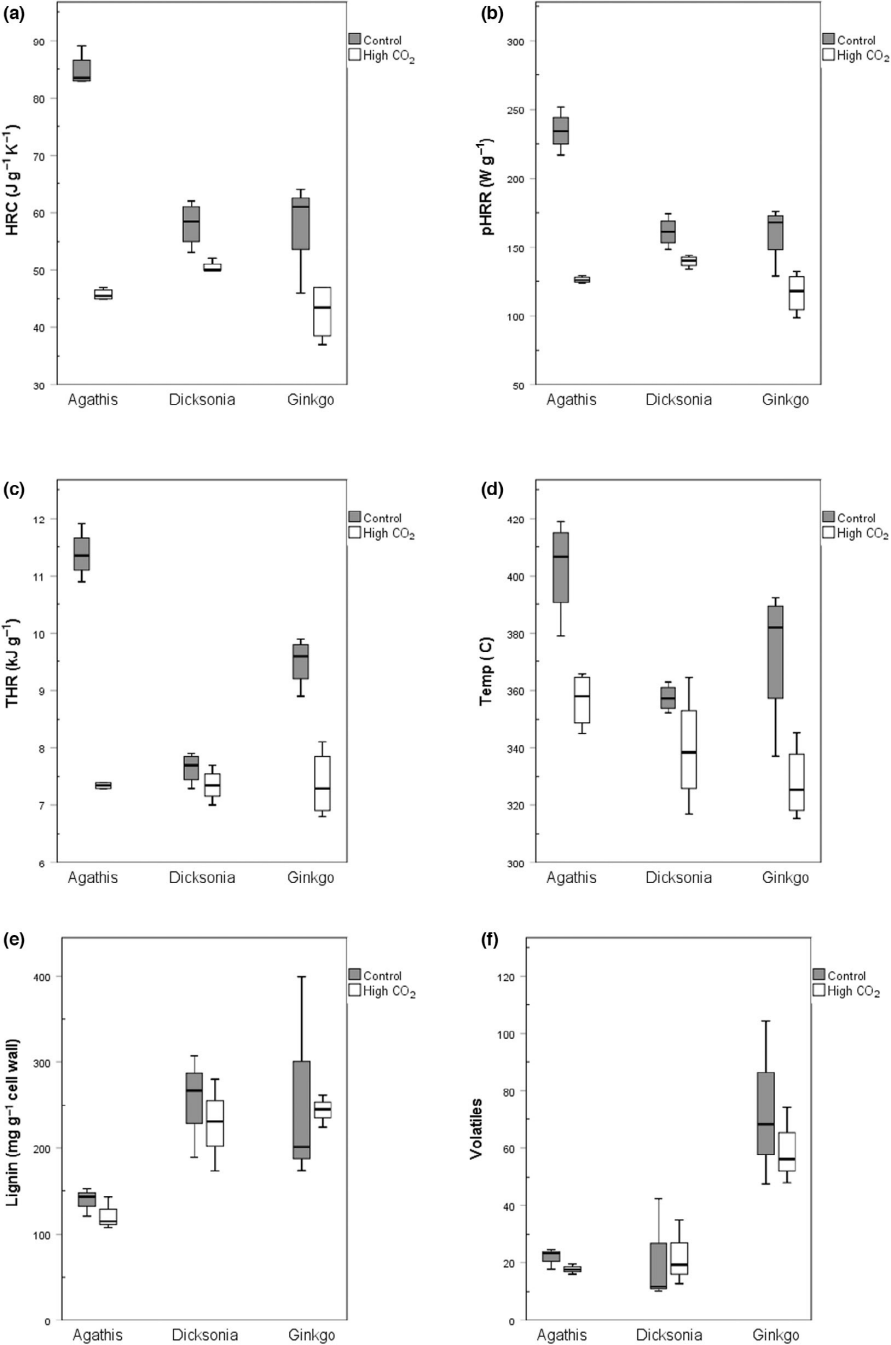
Flammability and chemistry of species under elevated CO_2_. Intrinsic flammability of *Dicksonia*, *Agathis* and *Ginkgo* was measured using a microcalorimeter. Heat release capacity (HRC; a), peak heat release rate (pHRR; b), total heat release (THR; c) and temperature of maximum decomposition (temp; d) were recorded. Lignin (e) was measured using the acetyl‐bromide method and volatile compounds (f) were measured using gas chromatography–mass spectrometry.

Both *A. australis* and *G. biloba* showed a decrease in volatile compounds (as detected on GC−MS) following 18 months of growth and development under elevated CO_2_ (Figs 1, 2). *Agathis australis* leaves grown in elevated CO_2_ showed a decrease in monoterpenes, which is consistent with observations for *Pinus sylvestris* leaves (Kainulainen *et al*., 1998). Similarly, *G. biloba* grown under elevated CO_2_ also showed a reduction in early‐eluting compounds (generally short‐chain fatty acids and alcohols rather than monoterpenes in *G. biloba*) compared to control atmosphere grown plants. The early‐eluting compounds represent the most volatile compounds, so a loss of these compounds may have an impact on leaf‐level flammability, by influencing the ease of ignition.

The three species all revealed lower leaf‐level flammability under elevated CO_2_ conditions, when measured using a microcalorimeter (see Fig. 2; Table 2). This trend was most apparent in *A. australis*, where the pHRR (peak intensity of flaming) under elevated CO_2_ was around half that of the control (126.0 J g^−1^ K^−1^ compared to 234.1 J g^−1^ K^−1^ in the control (*P* < 0.001)). Similar, but less extreme differences were seen in *D. antarctica* (*P* < 0.01) and *G. biloba* (*P* < 0.01). The THR describes the total energy release during combustion. The THR in all species and tests was higher in the control than under elevated CO_2_, with the exception of one test for *Dicksonia* where the THR was lower in the control compared to under elevated CO_2_ (*P* < 0.001 for *Agathis* and *Gingko*, and *Dicksonia P* > 0.05 at 0.176). The HRC (that indicates the resistance of the leaves to thermal degradation, where lower values of this measure report enhanced fire resistance), was also lower in elevated CO_2_ compared to the controls in all tests for each species (*P*‐value of < 0.01 for all species) (see Fig. 2; Table 2).

We found that the abundance of lignin in the leaves declined when grown under elevated CO_2_ conditions (Fig. 2e; Table S1). Here, the abundance of lignin decreased by 21% in *Dicksonia*, declining from a mean of 286.82 mg g^−1^ to 227.11 mg g^−1^ under elevated CO_2_; 19% in *Ginkgo*, decreasing from a mean of 300.40 mg g^−1^ to 242.93 mg g^−1^, and 25% in *Agathis* declining from a mean of 147.68 mg g^−1^ to 111.45 mg g^−1^. Interestingly however, a *P*‐test and Kruskal−Wallis test confirm that the lignin levels in all species showed no significant difference (*P* = 0.682 across all species, Kruskal−Wallis test, *n* = 26) between the two different atmospheric conditions.

### Changes in fire behaviour across the Triassic–Jurassic transition

The fuel models and environmental conditions inferred for Triassic plant beds 1 to 4 suggest that the surface rate of spread ranges between 4.1 m min^−1^ to 5.0 m min^−1^. Modelled fireline intensity ranges between 1583 kW m^−1^ to 2016 kW m^−1^ leading to scorch heights of between 17.0 m and 21.0 m (Table 3). The variations in fire behaviour estimated for these plant beds (Table 3) are driven by changes in inferred dominance of different leaf types in the litter (reflecting the composition of the overstory) and changes in the interpreted abundance of understory live fuels (in this case *D. antarctica*). This variation of surface fuel types influences the energy that the fuel can give to the fire (its heat content) which together account for the small changes in modelled fire behaviour between plant beds 1 through to 4. For example, Plant bed 1 contains fossil leaves of *Podozamites* and *Ginkgo*. Plant bed 2 similarly contains remains of *Podozamites* and *Ginkgo* as well as *Elatocladus*. Plant beds 3 and 4 contain remains of *Podozamites*, but are missing *Elatocladus* and *Ginkgo*. The modern analogue for *Podozamites* (*A. australis*) has a higher heat of combustion (HoC) value than *Ginkgo* and *Elatocladus*. This is reflected in the modelled fire behaviour outputs, which yield slightly higher rates of surface fire spread, greater scorch heights and increased fireline intensity within plant beds 3 and 4 when compared to plant beds containing litter fuels with lower HoC values, such as beds 1, 1.5 and 2.

**Table 3:** Results from behaveplus model runs adjusted for varying vegetation type, understory and leaf morphology (as described in the Materials and Methods section) and using a mid‐flame windspeed of 5.6 km h^−1^.

Vegetation‐fire phase (Belcher, 2016)	Plant bed number	Surface spread rate (m min^−1^)	Fireline intensity (kW m^−1^)	Scorch height (m)
Phase 1	Plant bed 1	4.4	1816	19.0
	Plant bed 1.5	4.1	1583	17.0
	Plant bed 2	4.4	1819	19.0
Phase 2	Plant beds 3 and 4	5.0	2016	21.0
	Plant bed 5A Scenario (a) ‘Control’	5.2	2206	22.0
	Plant bed 5A Scenario (b) Increased [CO_2_] effects on heat of combustion (HoC) + lignin only	3.5 (*4.3)	1003 (*1469)	12.0 (*16.0)
	Plant bed 5A Scenario (c) Increased [CO_2_] effects on HoC, lignin + 4°C increase	3.5 (*4.3)	1003 (*1469)	14.0 (*19.0)
	Plant bed 5A Scenario (d) Increased [CO_2_] effects on HoC, lignin + 11°C increase	3.5 (*4.3)	1003 (*1469)	18.0 (*24.0)
Phase 3 (plant bed 5B)	Scenario (a) ‘Control’	1.2	158	3.0
	Scenario (b) Increased [CO_2_] effects on HoC + lignin only	0.9 (*0.9)	98 (*96)	2.0 (*2.0)
	Scenario (c) Increased [CO_2_] effects on HoC, lignin + 4°C increase	0.9 (*0.9)	98 (*96)	2.2 (*2.2)
	Scenario (d) Increased [CO_2_] effects on HoC, lignin + 11°C increase	0.9 (*0.9)	98 (*96)	3.0 (*3.0)

* The same run but assuming no decrease in lignin content (following Soh *et al*., 2017).

Plant beds 1, 1.5, 2, 3, 4 and 5A model run using a broad‐leaved fuel model, that includes an understory TU5, where TU is a Timber Understory fuel model (see Scott & Burgan, 2005). Plant bed 5B run using narrow, needle leaf fuel model TL8, where TL is a timber litter fuel model (see Scott & Burgan, 2005) to capture changes in fuel morphology (Belcher *et al*., 2010; Belcher, 2016). Plant bed 5A and 5B model runs were conducted under four scenarios: (a) ‘Control’ conditions with no [CO_2_] or temperature change effects, only fuel changes; (b) increased [CO_2_] effects on heat of combustion (HoC) and lignin content only; (c) increased [CO_2_] effects on HoC, lignin content and a 4°C global temperature rise; (d) increased [CO_2_] effects on HoC, lignin content and an 11°C local summer temperature rise. For comparison, model run results where no increased [CO_2_] effects on lignin content, and therefore no change in decomposition rates (Soh *et al*., 2017) are also given for plant beds 5A and 5B and are denoted by (*number).

**Fig. 3:**
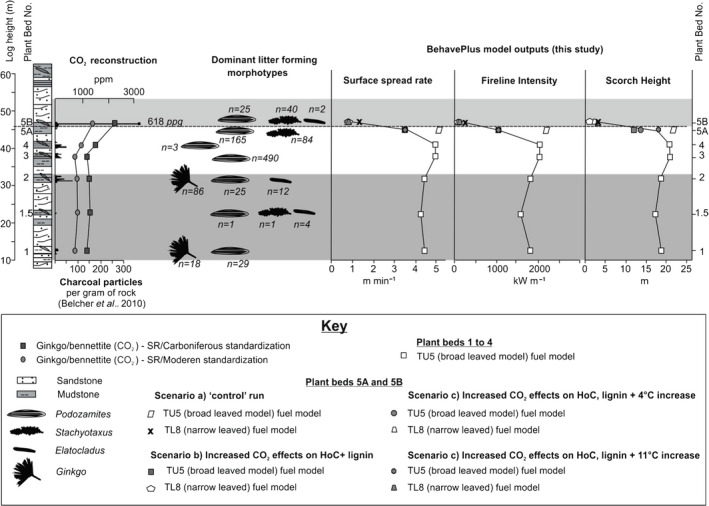
Modelled variations in palaeofire behaviour in the run‐up to the Triassic–Jurassic boundary transition at Astartekløft, East Greenland. Plant beds and lithology, reconstructed CO_2_ curve from Steinthorsdottir *et al*. (2011), plotted against total charcoal particles per gram of rock found in each plant bed (Belcher *et al*., 2010), dominant litter forming morphotypes (Belcher, 2016) and recorded numbers found in each plant bed (Belcher, 2016), and modelled fire behaviour outputs using behaveplus of surface spread rate, fireline intensity and scorch height for the different scenarios proposed.

Phase 2 (Triassic plant beds 3, 4 and 5A) (Belcher, 2016) is characterized by a hypothesized plant species richness decline when compared to those of Phase 1. Plant beds 3 and 4 being dominated by *Podozamites*, and bed 5A *Podozamites* and *Stachyotaxis*. Within this phase, there is also a hypothesized loss of the mid‐canopy and understory (McElwain *et al*., 2007; Belcher, 2016), represented by a halving of the live herbaceous fuel load in the fuel models for this phase (see the Materials and Methods section and Table S4 for information). Interestingly, this halving of the live herbaceous fuel load (in the fuel model) appears to have little impact on the modelled fire behaviour outputs, with surface spread rate, fireline intensity and scorch height remaining within a similar range to the phase 1 beds (Fig. 3). Between plant beds 3, 4 and 5A ‘control’, only fuel type is influencing the changes in fire behaviour modelled.

Plant bed 5A ‘control’ is modelled to have the fastest spread rate of 5.2 m min^−1^, as well as the highest fireline intensity of 2206 kW m^−1^ and scorch height of 22.0 m, whilst plant bed 5B ‘control’, which accounts for a change in leaf morphology only, is modelled to have the lowest spread rate of 1.2 m min^−1^; fireline intensity of 158 kW m^−1^ and scorch height of 3.0 m. By incorporating effects of increased CO_2_ on the HoC and lignin content into the models for plant beds 5A and 5B, spread rates, fireline intensity and scorch heights decrease, from 5.2 m min^−1^ to 3.5 m min^−1^, 2206 kW m^−1^ to 1003 kW m^−1^ and from 22.0 m to 12.0 m in plant bed 5A, respectively, and from 1.2 m min^−1^ to 0.9 m min^−1^; 158 kW m^−1^ to 98 kW m and from 3.0 m to 2.0 m in plant bed 5B, respectively. By increasing the air temperature for beds 5A and 5B, only scorch height is affected, increasing to 18.0 m for bed 5A and 3.0 m for bed 5B with an 11°C increase in air temperature. Removing the effects of lignin content (*number, Table 3), results in slightly higher spread rates, fireline intensity and scorch heights for bed 5A, whilst for bed 5B has no effect on spread rate or scorch height, but decreases the fireline intensity by 2 kW m^−1^ (Fig. 3). Here, *P*‐values of < 0.05 (largest being 0.01) confirm that model scenario’s (b), (c) and (d) run for plant bed 5A and scenario’s (a), (b), (c) and (d) for plant bed 5B are statistically significant when compared to the previous modelled fire behaviour for plants beds 1–4.

All results are shown in Table 3 and an example for an increased mid‐flame wind speed to 11.2 km h^−1^ in Table S5.

## Discussion

### Changes in leaf biochemistry

The evolution of secretory structures which contain terpenoid compounds is thought to have occurred in gymnosperms (Lange, 2015). Ferns have been documented to contain larger terpenoids (triterpenes and larger), but do not generally produce monoterpenes or sesquiterpenes (Lange, 2015). As such, *D. antarctica* was not found to contain many smaller volatile compounds in either atmospheric treatment (Figs 1, 2). In contrast to the gymnosperm species, *D. antarctica* leaves contained greater levels of long‐chain hydrocarbons under elevated CO_2_.

*Agathis australis* showed a marked decrease in flammability under elevated CO_2_. It has been suggested that volatile terpenoid compounds contribute to plant flammability (Varner *et al*., 2015; Dewhirst *et al*., 2020); however, lignin levels have been reported to increase under elevated CO_2_ (Lindroth, 2010). This was not replicated in the species in this study, which showed no significant difference in lignin levels between the two different atmospheric conditions. Therefore, the major decrease in flammability in *A. australis* is likely to be driven by a decrease in volatile compounds rather than lignin. As such, we hypothesize that the decrease in volatile compounds, particularly monoterpenes, in *A. australis* plays a role in the decrease in heat release. While the relatively short duration of this study may represent acclimation rather than adaptation of leaf biochemistry to elevated CO_2_, it is reasonable to assume that the adaptive response would be similar to the acclimation response. We found that the abundance of lignin in the leaves declined when grown in elevated CO_2_ (Fig. 2e; Table S1). Here, the abundance of lignin decreased by 21% in *Dicksonia*; 19% in *Ginkgo* and 25% in *Agathis*. Lignin content has been reported to have a variable response to elevated CO_2_; leaves of *Pinus sylvestris* (Overdieck & Fenselau, 2009) and *Betula pendula* Roth (Kostiainen *et al*., 2006) had decreased lignin content, whereas poplar (Luo & Polle, 2009), *Pinus densiflora* and *Quercus* (Park *et al*., 2018) foliage showed an increase in lignin. It may be that there are different responses between gymnosperms and angiosperms in regard to biochemical variations in response to elevated CO_2_. It has been shown that leaf litters containing leaves with lower lignin contents have a faster rate of decomposition than those with higher lignin contents (Wedderburn & Carter, 1999). We would therefore predict that as an example, *Podozamites*, one of the inferred dominant litter formers in the section, that continues to be present across the TJT, may have seen an increase in its ease of decomposition owing to a likely decrease in lignin due to rising CO_2_. Similarly, enhanced temperatures, when in conjunction with a rise in moisture, tend to speed up chemical reactions such that both would tend to lead to lower litter abundances being available to carry surface fires. We note however, that Soh *et al*. (2017) predicted greater leaf fuel on the ground, based on their estimates of an increase in leaf mass per area across the TJT at Astartekløft.

These results suggest that, if scaled up to the ecosystem level, periods of elevated CO_2_ and the resulting influence on heat release may be capable of altering fire behaviour. Hence, it could be hypothesized that CO_2_‐driven climate changes in the run‐up to the TJT may have induced both biochemical (this study) and morphological changes in fuel properties observed across plant beds 5A and 5B (Belcher *et al*., 2010; Belcher, 2016) capable of influencing fire behaviour.

The TJT provides a good case study for plant responses to a period of warming. Under current climate model projections, rising anthropogenic CO_2_ emissions are expected to increase annual temperatures by 3–6°C (Flato *et al*., 2013), similar to the estimated global average 3–4°C temperature rise across the TJT (McElwain *et al*., 1999). Hence plant responses during the TJT could provide a useful indicator to future changes in fire behaviour under a warming planet.

### Changing surface fire behaviour in the run‐up to the TJT

The fire behaviour estimates suggest that fire behaviour may have been more extreme prior to the increase in [CO_2_] in the latest Triassic (Table 3). Interestingly, model runs where a decrease in lignin content was not included lead to a smaller decline in surface spread rates fireline intensity and scorch heights when compared to the 5A control run, bringing spread rates, fireline intensity and scorch height to similar levels to pre ‘elevated [CO_2_]’ plant bed results. Here, surface spread rates declined by 17%, to 4.3 m min^−1^, fireline intensity by 33% to 1469 KW m^−1^ and scorch height by 27% to 16.0 m.

The latest Triassic plant bed 5B results run under ‘control’ conditions (scenario (a) – representing the change in observed leaf morphology to narrow leaved morphotypes and conifers, with limited understory only) show a marked decline in all parameters modelled compared to all previous ‘broad‐leaved’ Triassic plant bed model runs (Table 3; Fig. 3). Here, the average surface rate of spread was reduced from 4.4 m min^−1^ to 1.2 m min^−1^; average fireline intensity from 1708 kW m^−1^ to 158 kW m^−1^ and scorch height from 18.17 m to 3.0 m when compared to plant beds 1 to 5A ‘scenario (b)’ (elevated CO_2_ only). As shown in Belcher (2016), these results indicate that a change from broad‐leaved fuels with the presence of a fern understory to a narrow‐leaved conifer surface fuel with little understory has the ability to alter fire. Here, our results suggest a switch from a period of modelled fast‐moving surface fires, with high scorch heights and fireline intensity for plant beds 1 to 5A, to a period of reduced fire spread rates, fireline intensity and scorch heights during the vegetation change in plant bed 5B.

Model runs for plant bed 5B in which the 1 h load was reduced to represent the decline in lignin content and where the heat content was lowered to represent the change in biochemical flammability observed under ‘elevated [CO_2_] conditions’ (scenario (b)), results in a further observable decrease in all aspects of fire behaviour when compared to the bed 5B ‘control’ run. Here, the observed decrease in pHRR in all three species tested under elevated CO_2_ conditions, is reflected by the further 38% decrease in fireline intensity, 25% decrease in spread rate and 33% decline in scorch height in the ‘elevated [CO_2_]’ model run output compared to the control output. This decline in fireline intensity, coupled with a decline in surface fire spread and decline in scorch height observed within our ‘elevated [CO_2_]’ fire behaviour output suggests a period of reduced fire spread rate, declining to 0.9 m min^−1^; fireline intensity, declining to 98 KW m^−1^ and scorch height, declining to 2.0 m.

Increased mean annual global air temperature of +4°C (McElwain *et al*., 1999), coupled with elevated [CO_2_] influences on heat content and lignin content (scenario (c)), like that observed in plant bed 5A, increased the scorch height from 2.0 m to 2.2 m when compared to the elevated [CO_2_] only run (scenario (b)). Interestingly, there is no change in fireline intensity nor the surface spread rate. Moreover, by increasing the temperature to the maximum modelled mean summer temperature of 36°C (Huynh & Poulsen, 2005) (scenario (d)), scorch height further increases to 3.0 m for bed 5B, owing to drier canopy fuels. Interestingly, unlike in plant bed 5A, removing the effects of ‘elevated [CO_2_]’ on lignin contents has little effect on the modelled outputs, with no change in surface spread rate nor scorch height, and a decline of just 2 kW m^−1^ in fireline intensity throughout each of the ‘elevated [CO_2_]’ runs.

Our results show that the effects of increased CO_2_ on the heat of combustion and lignin content, appear to have a measurable influence on modelled fire behaviour; where with increasing atmospheric CO_2_, surface spread rate, fireline intensity and scorch height are reduced between 25–33%, 38–55% and 40–45%, respectively, in both plant beds 5A and 5B. Whilst altering the chemical parameters in the model runs (control vs [CO_2_] scenarios for both plant beds) did induce additional reductions in modelled surface spread rate, fireline intensity and scorch height by 3%, 3% and 11%, respectively, these shifts appear smaller than those induced by shifts in leaf morphology alone (control plant bed 5A vs control plant bed 5B) which displayed reductions of 77%, 93% and 86%, respectively. These results highlight that changes in plant chemical composition may likely have an impact on fire behaviour under rising CO_2_ concentrations, even where leaf morphology does not change, although, changes in leaf morphology do appear to be the dominant driver in altering modelled fire behaviour.

Current predictions for changes in fire regime under a warming climate, primarily focus on three aspects; (1) changes in fuel conditions, such as warmer, drier weather reducing moisture content of fuels, (2) the volume, structure and type of fuel available to burn, and (3) ignitions (Hessl, 2011; Flannigan *et al*., 2013; Keeley & Syphard, 2016). Using these, our modelling study results predict a general increase in rate of modelled fire spread, fire intensity and a lengthening of the fire season under a 2 × CO_2_ climate (Hessl, 2011), in Canadian boreal forests (de Groot *et al*., 2013), the Mediterranean (e.g. Mouillot *et al*., 2002), the western United States (Brown *et al*., 2004), Australia (Cary, 2002), and forest‐tundra (Abbott *et al*., 2016). Additionally, model simulations predict increased fuel consumption in boreal regions under increased CO_2_‐driven climates, with higher forest floor and crown fuel consumption rates in both western Canada and central Russia, although Russia is expected to see a much smaller increase when compared with current day climate conditions (de Groot *et al*., 2013).

However, the relationship between global warming and shifts in fire regimes is not simple, and has been shown to vary at regional levels depending on vegetation type and ignition probability. For example, Keeley & Syphard (2016) highlight that landscapes at lower elevation and latitudes show no or very little increase in fire activity, despite projected warmer, drier conditions due to limited ignitions. Additionally, Pausas & Paula (2012) conclude that fuel structure is more relevant than climate conditions in driving fire activity changes under Mediterranean climate conditions. Girardin *et al*. (2013) and Foster *et al*. (2022) highlight that changes in climate driven plant composition may in fact mitigate against the increase in fire frequency driven by climate change.

Keeley & Syphard (2016) suggest that the effects of rising CO_2_ on plants can further complicate fire regime predictions. For example, plants shift their optimum temperature for photosynthesis under elevated CO_2_ conditions, thus increasing productivity (Pausas & Paula, 2012). Furthermore, elevated CO_2_ concentrations have been found to increase water use efficiency, helping to protect some plants from drought, and increasing effective precipitation (Charney *et al*., 2016). Equally, fire events themselves led to increased water use efficiency in *Pinus halepensis* (Battipaglia *et al*., 2014), demonstrating a complex relationship between these factors.

Our results highlight that although climate and weather are known to play key roles in determining fire activity, and may increase modelled scorch heights under high CO_2_ conditions (e.g. our model scenario (c)); morphological and intrinsic fuel properties are equally important when considering the effects of rising CO_2_ on fire behaviour, and may actually lead to a reduction in fire spread rates and intensity in certain types of vegetation. Our results demonstrate that a coupled approach that incorporates variations in plant morphology and biochemistry, is essential if we are to predict both past and future shifts in fire regimes to changing climates and CO_2_ concentrations.

### Conclusion

Our results show that although leaf morphology may likely be the primary driving force behind changes in fire behaviour, CO_2_‐driven changes in leaf intrinsic flammability has an additional measurable impact on modelled fire behaviour that may be capable of lowering scorch heights by up to 45%. This coupled approach indicates that accounting for variations in plant morphological and chemical traits in models is essential when considering shifts in fire regimes. It is clear that interpretations based solely on the abundances of fossil charcoal will lead to errors in the understanding of palaeofire regimes and the palaeoecological impacts of fire. But this also indicates the importance of considering how current climatic change may influence the biochemical properties of plants that relate to flammability. Additional species and a range of plant groups should be assessed to determine whether or not CO_2_ enrichment has the possibility to alter biochemical plant traits and how this might impact on Earth’s future fire regimes.

## Author contributions

CMB designed the research. JCM, MH, CMB and RAD carried out the data collection and analysis. RAD undertook the chemical analyses. CMB undertook the flammability experiments. SJB undertook the fire behaviour modelling. SJB and RAD contributed equally to the writing of the manuscript and interpretation of the data. All authors contributed to writing and editing the manuscript.

## Supporting information


**Fig. S1** Agathis replicate GC profiles.
**Fig. S2** Dicksonia replicate GC profiles.
**Fig. S3** Ginkgo replicate GC profiles.
**Notes S1** Details of construction of fuel models for the TJT ecosystems at Astartekløft.
**Table S1** Flammability test results.
**Table S2** Volatile compound identification as detected by GC–MS.
**Table S3** Number of dominant litter‐forming conifer morphotypes within each plant bed.
**Table S4** Numerical description of the fuel model and parameters used for each of the plant beds modelled.
**Table S5** BehavePlus model run results adjusted for varying parameters.
Please note: Wiley Blackwell are not responsible for the content or functionality of any Supporting Information supplied by the authors. Any queries (other than missing material) should be directed to the *New Phytologist* Central Office.

## Data Availability

The data that support the findings of this study is openly available in Dryad, doi: 10.5061/dryad.tb2rbp033.
