# Supplementary material for: CO2 ‐induced biochemical changes in leaf volatiles decreased fire‐intensity in the run‐up to the Triassic–Jurassic boundary
Source: New Phytol. 2022 Jun 30;235(4):1442–54. doi: 10.1111/nph.18299 (PMC9545750; doi:10.1111/nph.18299)
Supplement: Supplementary file 1 — Fig. S1 Agathis replicate GC profiles. Fig. S2 Dicksonia replicate GC profiles. Fig. S3 Ginkgo replicate GC profiles. Notes S1 Details of construction of fuel models for the TJT ecosystems at Astartekløft. Table S1 Flammability test results. Table S2 Volatile compound identification as detected by GC–MS. Table S3 Number of dominant litter‐forming conifer morphotypes within each plant bed. Table S4 Numerical description of the fuel model and parameters used for each of the plant beds modelled. Table S5 BehavePlus model run results adjusted for varying parameters. Please note: Wiley Blackwell are not responsible for the content or functionality of any Supporting Information supplied by the authors. Any queries (other than missing material) should be directed to the New Phytologist Central Office. [file NPH-235-1442-s001.pdf]

CO_2-induced biochemical changes in leaf volatiles decreased fire intensity in the run-up to the Triassic-Jurassic boundary

Sarah J. Baker*, Rebecca A. Dewhirst*, Jennifer C. McElwain, Matthew Haworth and Claire M. Belcher

* both authors contributed equally to this work

Article Acceptance: 22nd April 2022

	Sample Name	Species	Treatment	Lignin mg/g cell wall	HR capacity J/g-K	Peak HRR W/g	Total HR kJ/g	Temp °C	Total volatiles
	AA 1A	Agathis	Control	143.1818	89	252	11.4	418.7	23.2300
	AA 2A	Agathis	Control	152.1739	84	232.7	10.9	411	24.7339
	AA 3D	Agathis	High CO_2	115	45	125.1	7.4	344.9	17.6366
	AA 5D	Agathis	High CO_2	107.8947	46	126.9	7.3	363.2	15.9886
	D1D	Dicksonia	High CO_2	280.1666	52	143.7	7.3	341.6	35.0306
	D2A	Dicksonia	Control	266.8181	60	163.7	7.6	355.6	10.2189
	D3A	Dicksonia	Control	306.8181	57	158.9	7.9	352.3	42.2373
	D3D	Dicksonia	High CO_2	174.0476	50	133.9	7.4	335.1	12.7796
	GB 24A	Ginkgo	Control	201.3043	46	128.9	8.9	377.6	104.379
	GB 28A	Ginkgo	Control	399.5	61	169.3	9.5	386.4	47.4083
	GB 5D	Ginkgo	High CO_2	261.8666	47	124.8	8.1	315.3	56.2321
	GB 8D	Ginkgo	High CO_2	224	40	111.3	7	320.8	48.0052
**Replicates**	AA1A 2	Agathis	Control	-	83	235.4	11.9	402.2	-
	AA2A 2	Agathis	Control	-	83	217	11.3	379	-
	AA3D 2	Agathis	High CO_2	-	47	129.2	7.4	352.8	-
	AA5D 2	Agathis	High CO_2	-	45	124	7.3	365.7	-
	D2A 2	Dicksonia	Control	-	53	147.9	7.3	359	-
	D3A 2	Dicksonia	Control	-	62	174.3	7.8	362.9	-
	D3D 2	Dicksonia	High CO_2	-	50	138.9	7.7	316.9	-
	D1D 2	Dicksonia	High CO_2	-	50	141.1	7	364.4	-
	GB28A 2	Ginkgo	Control	-	64	175.9	9.7	392.2	-
	GB24A 2	Ginkgo	Control	-	61	166.6	9.9	337.1	-
	GB5D 2	Ginkgo	High CO_2	-	47	132.2	7.6	345.2	-
	GB8D 2	Ginkgo	High CO_2	-	37	98.4	6.8	330.3	-

	Sample	Species	Treatment	Lignin mg/g cell wall	HR capacity J/g-K	Peak HRR W/g	Total HR kJ/g	Temp °C	Total volatiles
**Means**	AA A	Agathis	Control	147.67785	84.75	234.275	11.375	402.725	21.91454636
	AA D	Agathis	High CO_2	111.44735	45.75	126.3	7.35	356.65	17.69931717
	GB A	Ginkgo	Control	300.40215	58	160.175	9.5	373.325	73.31934919
	GB D	Ginkgo	High CO_2	242.9333	34.2	93.34	5.9	262.32	59.51809417
	D A	Dicksonia	Control	286.8181	58	161.2	7.65	357.45	21.39254222
	D D	Dicksonia	High CO_2	227.1071	50.5	139.4	7.35	339.5	22.33865734

Table S1: Results from flammability tests, taken from leaves grown under control and high CO_2 conditions.

Label	Identity	Structure	Compound class
**1**	Trimethyl pentadecanone	$C_{18}H_{36}O$	Isoprenoid ketone
**2**		$C_{20}H_{32}$	diterpene
**3**	kaur-16-ene	$C_{20}H_{32}$	diterpene
**4**			unknown
**5**	methyl-hexacosane	$C_{27}H_{56}$	long-chain hydrocarbon
**6**			long-chain hydrocarbon
**7**			long-chain hydrocarbon
**8**			long-chain hydrocarbon
**9**			long-chain hydrocarbon
**10**			long-chain hydrocarbon
**11**			unknown
**12**	3-Nonen-1-ol	$C_9H_{18}O$	fatty alcohol
**13**	heptanoic acid	$C_7H_{14}O_2$	fatty acid
**14**	myristic acid	$C_{14}H_{28}O_2$	fatty acid
**15**	eicosyne	$C_{20}H_{38}$	hydrocarbon
**16**	eicosyne	$C_{20}H_{38}$	hydrocarbon
**17**			unknown
**18**	juvabione	$C_{16}H_{26}O_3$	sesquiterpene
**19**	kaur-15-ene	$C_{20}H_{32}$	diterpene
**20**	elaidic acid	$C_{18}H_{34}O_2$	trans fatty acid
**21**	stearic acid	$C_{18}H_{36}O_2$	fatty acid
**22**	sclareol	$C_{20}H_{36}O_2$	diterpene alcohol
**23**	phytol acetate	$C_{22}H_{24}O_2$	diterpene alcohol
**24**			
**25**	cembrene	$C_{20}H_{32}$	diterpene
**26**	tetramethylheptadecanolide	$C_{21}H_{40}O_2$	fatty acid
**27**			unknown
**28**	pentadecyl phenol	$C_{21}H_{36}O$	phenolic lipid
**29**			long-chain hydrocarbon
**30**			unknown
**31**			unknown
**32**			unknown
**33**			unknown
**34**			unknown
**35**	hentriacontane	$C_{31}H_{64}$	long-chain hydrocarbon
**36**			long-chain hydrocarbon
**37**			unknown
**38**	1-heptacosanol	$C_{27}H_{56}O$	fatty alcohol
**39**			unknown
**40**		$C_{10}H_{16}$	monoterpene
**41**	a-pinene	$C_{10}H_{16}$	monoterpene
**42**	camphene	$C_{10}H_{16}$	monoterpene
**43**	b-pinene	$C_{10}H_{16}$	monoterpene
**Label**	**Identity**	**Structure**	**Compound class**

**44**	verbenone	$C1_0H_{14}O$	monoterpene (oxygenated)
**45**	a-copaene	$C_{15}H_{24}$	sesquiterpene
**46**		$C_{15}H_{24}$	sesquiterpene
**47**		$C_{15}H_{24}$	sesquiterpene
**48**	g-cadinene	$C_{15}H_{24}$	sesquiterpene
**49**	spathulenol	$C_{15}H_{24}O$	sesquiterpene alcohol
**50**	17-norkaur-15-ene	$C_{20}H_{32}$	diterpene
**51**		$C_{20}H_{32}$	diterpene
**52**			unknown
**53**			long-chain hydrocarbon
**54**			long-chain hydrocarbon

Table S2: Detailed identification of volatile compounds as detected by GC-MS.

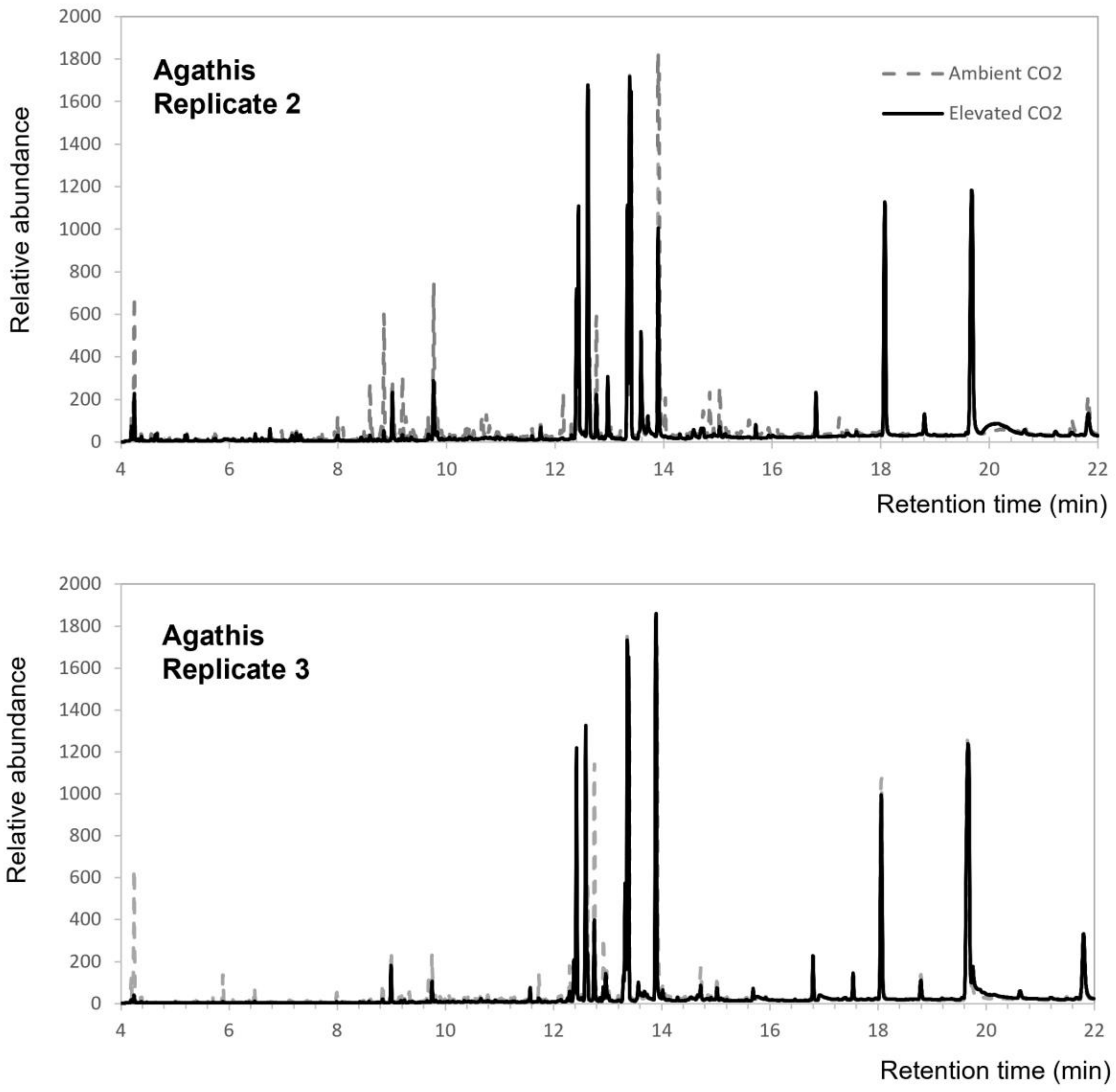

Figure S1: Agathis replicate GC profiles

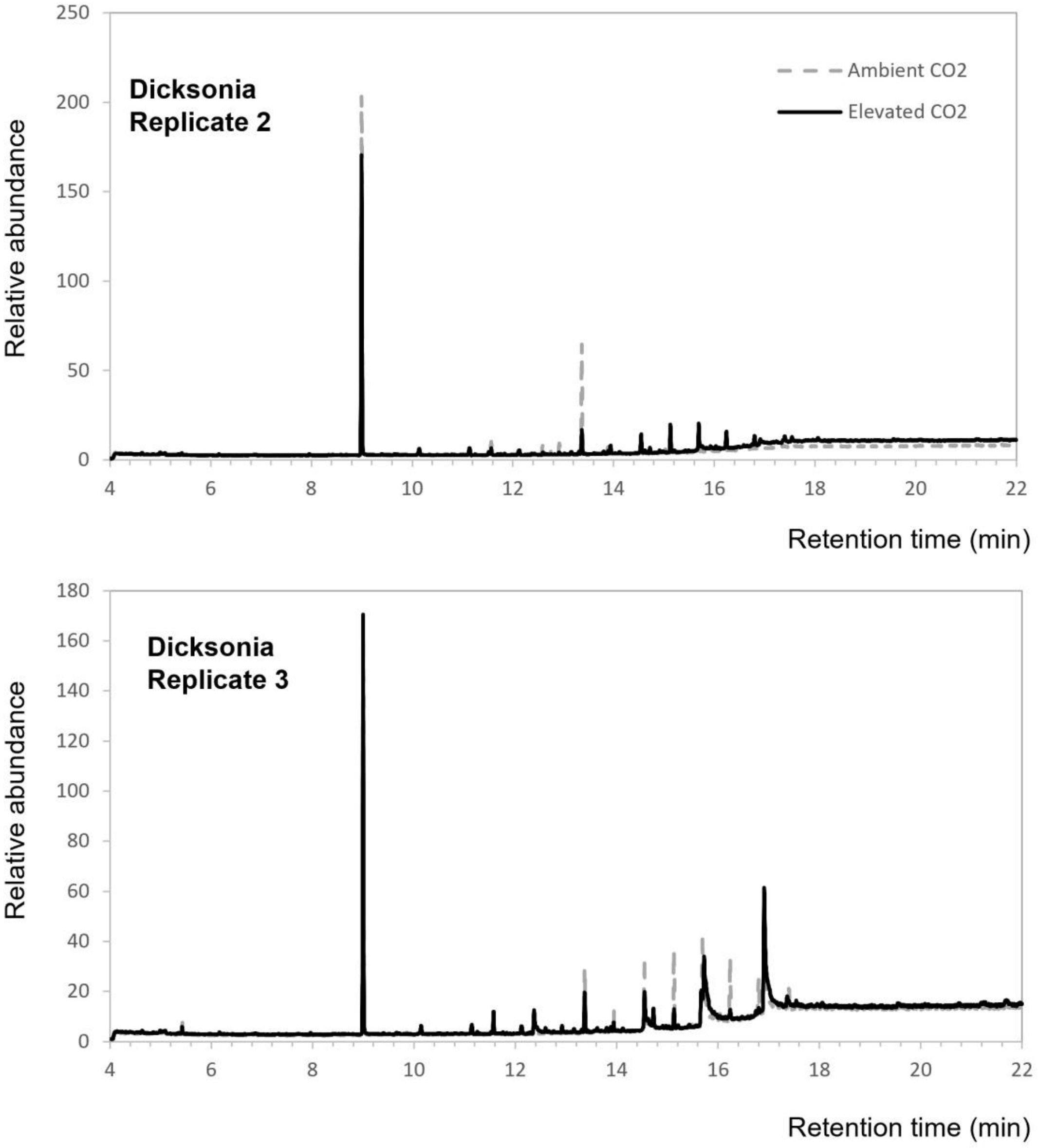

Figure S2: Dicksonia replicate GC profiles

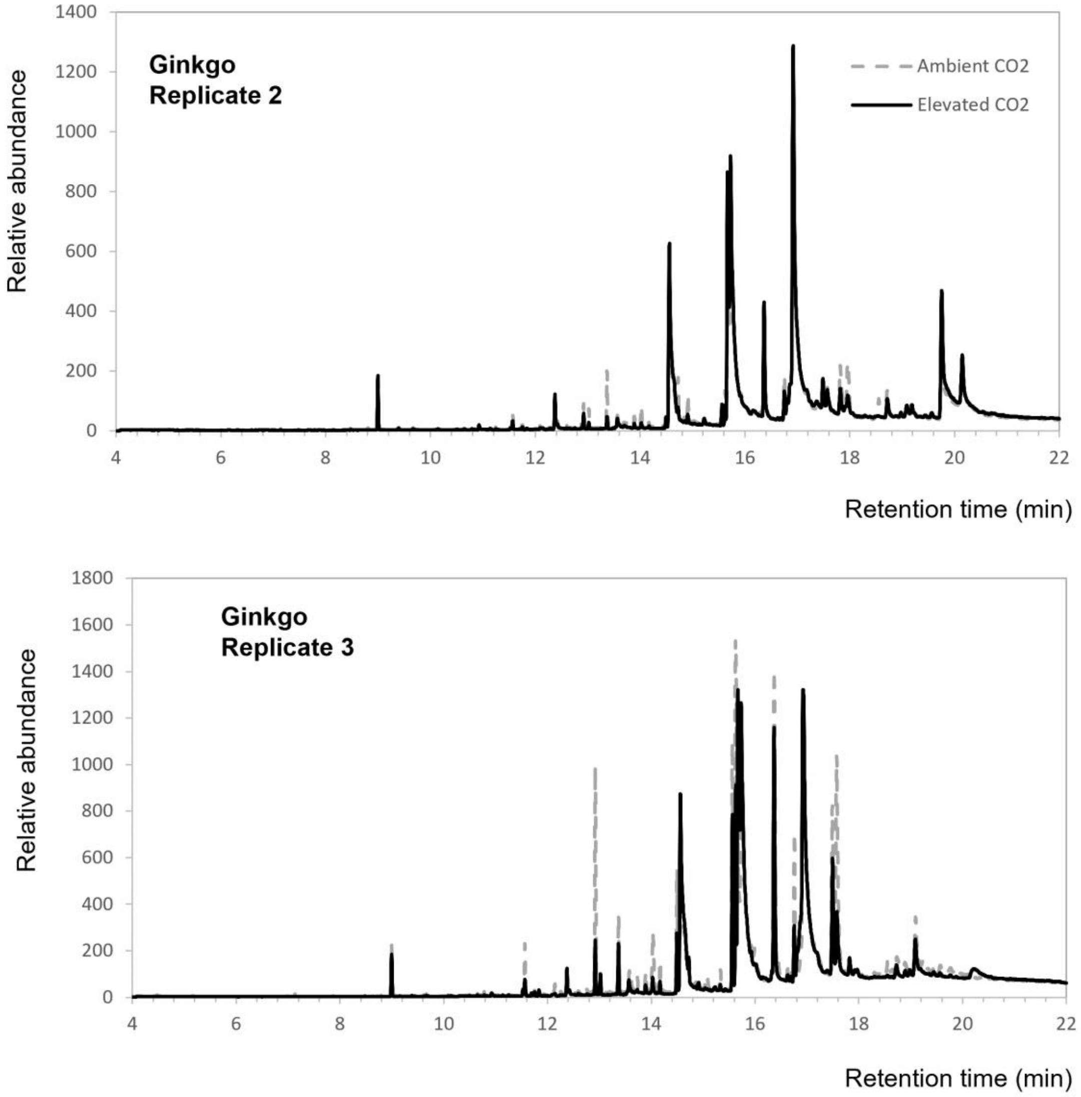

Figure S3: Ginkgo replicate GC profiles

Notes S1

Constructing Fuel Models for the TJT Ecosystems at Astartekløft.

Each fuel model requires a series of input parameters consisting of descriptors of the fuel; dead 1 hr, 10 hr and 100 hr fuel loads, live herbaceous fuel load, live woody fuel load, 1 hr surface area to volume ratio, fuel bed depth, dead fuel moisture of extinction and dead and live fuel heat contents. These define the nature and amount of each fuel class available to a fire (fuel load) and the bulk density of the fuel. The dead 1 hr, 10 hr and 100 hr time lag

fuels, are based on their ability to lose moisture and reach equilibrium moisture content (Scott and Burgan, 2005; Belcher and Hudspith, 2017). Hence, 1 hr fuel loads represent dead fine fuels of with a pre-defined diameter of <0.64 cm; dead 10 hr represent fuels up to 7.62 cm diameter, and dead 100 hr fuel loads represent larger fuels with diameters of >7.62 cm. The dead and live fuel in the fuel model must also have a 'heat content' assigned to it, describing the amount of energy the fuel can provide to the fire. BEHAVEPLUS also requires environmental parameter inputs, such as slope steepness, mid-flame wind speed (upslope), air temperature and fuel moisture.

BEHAVEPLUS has fifty-three pre-defined fuel models, and similarly to (Belcher and Hudspith, 2017), we utilize a set of these fuel models but make adjustments to them to describe the TJT fuel types. We adjusted the TU5 fuel model (Scott and Burgan, 2005), representative of broad-leaved, timber understory, to represent the broad-leaved fuels observed in the Triassic stage at Astartekløft, of ferns, cycads and bennettitales and broad-leaved gymnosperms. The TL8 model, representative of a moderate load of long-needle conifer litter, with little understory, was adjusted to represent the change in vegetation to narrow-leaved conifer dominated ecosystem of the transition bed 5B at Astartekløft. Adjustments were made to the dead fuel load, the live fuel load and the moisture of extinction, the latter of which was calculated based on the oxygen content of the Triassic/Jurassic atmosphere using the Watson and Lovelock (2013) moisture of extinction (M_{ex}) equation of $M_{ex} = 8O_2 – 128$, and the modelled atmospheric O_2 estimate of the Triassic/Jurassic atmosphere of 21% (Lenton et al., 2018). The heat content of both the dead and live fuels were adjusted based on our experiments using leaves grown under control and elevated [CO_2] conditions and for the hypothesized temperature changes across the T-J transition. These changes and calculations are detailed below, in Table S3 and Table S4.

Static Fuel Parameters

The vegetation during phases 1 and 2 (plant beds 1; 1.5, 2, 3, 4 and 5A) at Astartekloft consisted of broadleaved fuels such as ferns, cycads, bennettites and the broadleaf gymnosperms *Podozamites, ginkgo, Stachyotaxus and Elatocladus* (Belcher et al., 2010; Belcher, 2016). These would have provided a surface litter fuel bed consisting of broad-leaves, hence fuel model TU5 representative of 'broad-leaved, low load timber shrub' (Scott and Burgan, 2005) was chosen.

The TU5 model has a pre-defined dead fuel load for 1hr fuels of 8.97 t/ha; a 10-hr fuel of 8.97 t/ha and a 100-hr fuel of 6.70 t/ha and pre-defined live woody fuel load value of 6.73 t/ha. TU5 represents a forest litter with shrub/small tree understory, and thus provides a good similarity to the sub-canopy of tree ferns, growing alongside a canopy of woody broad-leaved gymnosperms suggested for the Astartekløft site. Hence, the pre-defined TU5 fuel loads and relative surface area to volume ratios (SA:V) for 1-hr; live herbaceous and live woody were retained. The fuel bed depth however is altered to 0.9 m based on the typical understory fern height (Ainsworth et al., 2010; Belcher and Hudspith, 2017). These values were kept as constants throughout the runs representing plant beds 1; 1.5, 2, 3, 4, and 5Acontrol (Table S4).

The vegetation during phase 3 is that of latest Triassic plant bed 5B, which indicates a change in vegetation to a narrow-leaved conifer dominated ecosystem (plant bed 5B, phase 3), hence fuel model TL8 was chosen. TL8 represents a moderate load of long-needle conifer litter, with little understory, and has a pre-defined 1-hr dead fuel load of 13 t/ha; 10-hr dead fuel load of 3.14 t/ha and 100-hr dead fuel load of 2.47 t/ha which, as in the Triassic TU5 model runs, are retained. Similarly the pre-defined 1-hr SA:V; live herbaceous SA:V and live woody SA:V are retained.

The heat content of the live surface fuels (live fuel heat content Table S4) was maintained at 15390 (kJ/kg^{-1}) in the following plants bed runs: 1, 1.5, 2, 3, 4, 5A control and 5B control. This value for the heat content is based on that from the combustion of *Dicksonia antarctica* fronds and was taken from the experiments of (Belcher and Hudspith, 2017). *Dicksonia antarctica* was selected as being a representative nearest living equivalent for the tree ferns and ferns that inhabited the subcanopy and understory at Astarteloft, in East Greenland during this time period (McElwain et al., 2007).

Static environmental parameters

As our aim with this study was to assess how climate-driven changes in leaf-biochemistry and leaf-level flammability would influence fire behaviour, we kept the parameters for slope steepness and fuel moisture constant in all model runs. Following Belcher and Hudspith (2017), a relatively shallow slope of 30% and live fuel moistures of 100% and dead fuel moistures of 8% were assigned. This live fuel moisture is representative of mature foliage and is also the suggested moisture content for BEHAVEPLUS when no other fuel moisture information is available (Belcher and Hudspith, 2017). A dead fuel moisture of 8% was chosen based on the Fire Behaviour Field Reference Guide, Fosberg Model estimates for dead fuel moisture content during summer months, under a relative humidity of 50 – 54% and a temperature of ~21°C to 42°C (covering the range of estimated air temperatures for the TJT), (accessed via: http://www.fbfrg.org/fuel-moisture/3-1-fuel-moisture-background-resources).

Variable fuel parameters

Assigning Heat Contents to each Fuel Model

To represent the changes in the heat content in the fuel models that represent the plant beds, we take the changes in the dominant litter morphotypes that would have provided the surface fuel for the fires. We selected the same four dominant canopy- and litter-forming morphotypes as that used in (Belcher, 2016) analysis of Astartekløft; *Elatocladus, Stachyotaxis, ginkgos* and *Podozamites*. In order to provide the heat contents of the fossil conifers we used either living morphological equivalents or nearest living relatives. For the broad-leaf conifer genus *Podozamites,* we use the modern *Agathis australis* as the morphological equivalent; for *Stachyotaxus*, the modern *Sequoia sempervirens*, and for *Elatocladus,* a needle/narrow leaf conifer, the species *Abies concolor* is used. We used *Ginkgo biloba* as the nearest living relative of the Ginkgo's at Astartekløft. For the fern understory, *Dicksonia antarctica* is used. The remainder of the vegetation at Astartekløft is captured within the fuel loads, SA:V etc, as defined above.

We took the heat of combustion (HoC) (MJ kg^{-1}) for each of the above mentioned species from Belcher (2016) and Belcher and Hudspith (2017) to inform the heat content of the models. The standard heat contents defined in BehavePlus do not well capture the type of fuels that existed during the Mesozoic, in fact today they generally only describe a range of fire prone ecosystems. BehavePlus has a minimum heat content limit based on ecosystem's today however, the heat contents for the morphological equivalents and nearest living relatives were below the minimum allowed. Hence all were scaled by adding a constant to each heat content - a similar approach was used by Simpson et al., (2016) when modelling modern savanna grass fires using BehavePlus. No data was available for *Ginkgo biloba* therefore we took its control value as being the minimum permissible in BehavePlus.

The relative percentages of the number of the dominant litter-forming fossil leaf morphotypes found within each plant bed (Table S3, and Belcher, 2016) were then used to weight the heat of combustion for each plant bed to reflect which genera were dominant. For

example, plant bed 1 contains 29 *Podozamites* and 18 *Gingkos*, hence 62% of the major canopy/litter forming plants are taken to be *Podozamites*, and 38% *Ginkgo* (Table S3). Therefore, plant bed 1 (as an example) was given a heat content value of 13473 kJ kg^{-1} based on a weighting of 62% on the HoC of *Podozamites* modern equivalent *Agathis australis* of 13171 kJ kg^{-1} and 38% weighting on the HoC of *Ginkgo* estimated at 13967 kJ kg^{-1}. The translation of the abundance percentages to average adjusted heat contents for each plant bed are shown in Table S3.

Finally, in order to represent the impact of elevated CO_2 in the heat content within each fuel model the heat content for the high [CO_2] runs (plant Bed 5A and 5B) were altered proportionally according to the change indicated by our plants grown under elevated CO_2 conditions (*Agathis australis; Dicksonia antarctica, Ginkgo biloba*). The heat contents were adjusted according to the percentage change, for example, *Agathis australis* saw a 35% THR reduction from a mean of 11.375 kJg^{-1} under control conditions compared to a mean of 7.35 kJg^{-1} under elevated CO_2; *Dicksonia antarctica* saw a 4% reduction in mean THR, and *Ginkgo biloba* a 22% reduction. The percentage reductions in total heat release are then applied to the relative measured heat of combustion from Belcher (2016) and Belcher and Hudspith (2017). The adjustment is shown in Table S3 and all fuel models in Table S4.

Fuel load variations

For the live herbaceous fuel load input, it is assumed that the fern understory represents the live ‘herbaceous’ fuel load, whilst the leaves/shoots shed from the canopy fuels are taken to be the dead litter fuel load. Hence, for the live herbaceous fuel load, a value of 6 % was chosen to represent the fern understory present in Triassic plant beds 1, 1.5 and 2 (phase 1) (Table S4). For plant beds 3, 4 and 5A the loss of the sub-canopy of cycads and bennettites (McElwain et al., 2007), is represented in the fuel model by a halving of the live herbaceous

fuel load (to 3% Table S4). To then represent the loss of much of the understory in plant bed 5B a value of 1 is given to the live fuel load, and the fuel bed depth is reduced to 0.09 m to represent a dominant conifer litter bed depth (see Table S4).

To represent the apparent decline in content of the leaves and its influence on the rate of decomposition of the leaf litter, we also adjusted the 1 hr fuel load in the model runs that accounted for enhanced CO_2. Both 1 hr fuel loads were decreased by 25% (in line with the 25% reduction observed in *Agathis* australis under elevated [CO_2] conditions, as *Podozamites* is present in both 'elevated [CO_2]' plant beds) to capture a scenario where the litter fuels would decompose more rapidly, due to decreased lignin content and enhanced temperatures. The 1hr fuel load in the bed 5A[CO_2] and [CO_2] temperature runs was adjusted from 8.97 tonnes per hectare to 6.72 tonnes per hectare. The 1 hr fuel load in the bed 5B[CO_2] and [CO_2] temperature runs were adjusted from 13 tonnes per hectare to 9.75 tonnes per hectare.

To model the potential effect of elevated [CO_2] on fire behaviour, the percentage reductions observed within our measured THR were applied to the relative measured heat of combustion where, for example, *Agathis australis* heat of combustion under control conditions was 13171 KJ Kg^{-1}, with a 35% loss resulting in 8561.15 KJ Kg^{-1} (see Table 1, Table S3, S4), and *Dicksonia antarctica* reduces from 15390 kJ kg^{-1} (Belcher and Hudspith, 2013) under control to 14744 kJ kg^{-1} under elevated [CO_2] conditions.

Variable Environmental Parameters for Different Scenarios

All models were run at two wind speeds one a gentle mid-flame windspeed of 5.6km/h and double the mid-flame wind speed of 11.2km/h (see Table S5). Extra model runs were also conducted for plant beds 5A and 5B to compare how fuel changes alone would influence fire and how CO_2 changes on top of these would influence fire. Hence a run labelled 5Acontrol or 5Bcontrol was run with no 'elevated [CO_2] influence' on the heat content parameters, no

effect of a decrease in lignin abundance influencing the 1hr fuel load, and no air temperature rise to provide a 'control' run which just captures the change in vegetation. Runs were also conducted under the influence of elevated CO_2 for no temperature change (5A[CO_2] 5B[CO_2]), a 4°temperature rise, and an 11°C temperature rise (representing the modelled summer temperature for East Greenland (McElwain et al., 1999; Huynh and Poulsen, 2005)) to capture the effects of rising temperature and CO_2 on modelled fire behaviour.

	Vegetation type	Plant bed number						
		**1**	**1.5**	**2**	**3**	**4**	**5A**	**5B**
		**Phase 1**			**Phase 2**			**Phase 3**
**Canopy Elements as % of dominant canopy elements burning as litter**	*Podozamites*	29 (62%)	1 (10%)	25 (20%)	490 (100%)	3 (100%)	165 (66%)	25 (37%)
	Stachyotaxis	0 (0%)	1 (10%)	0 (0%)	0 (0%)	0 (0%)	84 (34%)	40 (60%)
	Ginkgo	18 (38%)	0 (0%)	86 (70%)	0 (0%)	0 (0%)	0 (0%)	0 (0%)
	Elatocladus	0 (0%)	4 (80%)	12 (10%)	0 (0%)	0 (0%)	0 (0%)	2 (3%)
	Totals	**58 (100%)**	**6 (100%)**	**123 (100%)**	**490 (100%)**	**3 (100%)**	**249 (100%)**	**67 (100%)**

**Modern Equivalents of canopy elements and their heat of combustion ($KJKg^{-1}$)**			
Podozamites – Agathis australis	*Stachyotaxis – Sequoia sempervirens*	*Ginkgo – Ginkgo biloba*	*Elatocladus – Abies concolor*
13171 (1)	16640 (1)	13967 (*)	10850 (2)

**Dead fuel heat contents per plant bed based on weightings of modern equivalents and dominant canopy element %**								
	Vegetation type	Plant bed number						
		**1**	**1.5**	**2**	**3**	**4**	**5A**	**5B**
		**Phase 1**			**Phase 2**			**Phase 3**
Heat content calculations based on weighting of the HoC of modern equivalents and the % dominance of canopy elements in plant beds	*Agathis (Podozamites)*	(13171 x 0.62)	(13171 x 0.10)	(13171 x 0.20)	(13171 x 1.00)	(13171 x 1.00)	(13171 x 0.66) *(*or 8561*)	(13171 x 0.37) *(*or 8561*)
	Sequoia (Stachyotaxis)	0	(16640 x 0.10)	0	0	0	(16640 x 0.34)	(16640 x 0.60)
	Ginkgo (Ginkgo)	(13967 x 0.38)	0	(13967 x 0.70)	0	0	0	0
	Abies (Elatocladus)	0	(10850 x 0.80)	(10850 x 0.10)	0	0	0	(10850 x 0.03)
	Total weighted Heat Contents ($KJKg^{-1}$)	**13473**	**11661**	**13496**	**13171**	**13171**	**14350**	**14857**
***Effects of reduced HoC under elevated CO_2 conditions**	Constant HoC added *($KJKg^{-1}$)*	4609.9	4609.9	4609.9	4609.9	4609.9	4609.9	4609.9
	Total Heat Contents used in fuel models *($KJKg^{-1}$)*	**18083**	**16271**	**18106**	**17781**	**17781**	**18960** ***14350**	**19467** ***14857**

Under elevated CO_2 conditions (beds 5A and 5B only). Under elevated CO_2, Agathis (Podozamites) saw a 35% decline in total heat release, resulting in a new Heat Content of 8561 $KJKg^{-1}$.

**Understory fern dominants as occurrence in each plant bed**							
Vegetation type	Plant bed number						
	**1**	**1.5**	**2**	**3**	**4**	**5A**	**5B**
	**Phase 1**			**Phase 2**			**Phase 3**
Doratophyllum	3	20	0	1	60	0	1
Pterophyllum	41	3	44	6	271	1	0

Table S3 – Number of dominant litter-forming conifer morphotypes found within each plant bed that were used to inform the HoC for the surface fuels in each fuel model and dominant understory fern abundances (taken from Belcher et al. (2010) SI table 41561_2010_BFngeo871_MOESM293_ESM.xls; https://static-content.springer.com/esm/art%3A10.1038%2Fngeo871/MediaObjects/41561_2010_BFngeo871_MOESM293_ESM.xls), and their calculated percentage representation of the four dominant litter-forming types found in each plant bed (in brackets).
(1) Belcher and Hudspith, 2017, (2) Belcher et al., 2010, SI table rstb20150163supp2.xlsx); (*) No data was available for *Ginkgo biloba* therefore we took its control value as being the minimum permissible in BehavePlus.

Fuel parameter	Plant bed number								
	**1**	**1.5**	**2**	**3**	**4**	**5A control**	**5A[CO_2]**	**5B control**	**5B[CO_2]**
Fuel model	TU5	TU5	TU5	TU5	TU5	TU5	TU5	TL8	TL8
1-h fuel load (t ha^{-1})	8.97	8.97	8.97	8.97	8.97	8.97	6.72	13.00	9.75
10-h fuel load (t ha^{-1})	8.97	8.97	8.97	8.97	8.97	8.97	8.97	3.14	3.14
100-h fuel load (t ha^{-1})	6.73	6.73	6.73	6.73	6.73	6.73	6.73	2.47	2.47
Live herbaceous fuel load (t ha^{-1})	6	6	6	3	3	3	3	1	1
Live woody fuel load (t ha^{-1})	6.73	6.73	6.73	6.73	6.73	6.73	6.73	0	0
1-h SA:V (m^2 m^{-3})	4921	4921	4921	4921	4921	4921	4921	5906	5906
Live herbaceous SA:V (m^2 m^{-3})	5906	5906	5906	5906	5906	5906	5906	5906	5906
Live woody SA:V (m^2 m^{-3})	2461	2461	2461	2461	2461	2461	2461	5249	5249
Fuel bed depth (m)	0.9	0.9	0.9	0.9	0.9	0.9	0.9	0.09	0.09
Dead fuel moisture of extinction (%)	40	40	40	40	40	40	40	40	40
Dead fuel heat content (kJ kg^{-1})*	18083	16271	18106	17781	17781	18960	14350	19467	14857
Live fuel heat content (kJ kg^{-1})*	15390	15390	15390	15390	15390	15390	14774	15390	14774
Dead fuel moisture	8	8	8	8	8	8	8	8	8
Live fuel moisture	100	100	100	100	100	100	100	100	100
Air temperature (°C)	25	25	25	25	25	25	25, 29, 36	25	25, 29, 36

Table S4 – Numerical description of the fuel model and parameters used for each of the modelled Triassic plant beds. *Values derived from the weighting of HoC measurements using abundance percentages of fossil leaves found in the respective plant beds (Belcher et al., 2010). Line between beds 5A and 5B denotes fuel change from broad-leaved fuel model TU5 to narrow-leaved morphologies, represented by narrow-leaf fuel model TL8. Note, the change in 1hr fuel load between the ‘control’ runs and ‘elevated CO_2’ runs to account for the percentage change observed in lignin abundance of 25% found in *Agathis* under elevated CO_2.

**Vegetation-fire phase (Belcher et al., 2016)**	**Plant Bed number**	**Surface spread rate** (m/min)	**Fireline Intensity** (kW/m)	**Scorch Height** (m)
Phase 1	Plant bed 1.	9.0	3714	28.0
	Plant bed 1.5.	8.4	3238	25.0
	Plant bed 2.	9.0	3720	28.0
Phase 2	Plant bed 3 and 4	10.1	4050	30.0
	Plant bed 5A<br>**Scenario a)** 'Control'	10.5	4431	32.0
	Plant bed 5A<br>**Scenario b)** Increased [CO_2] effects on HoC + lignin only	7.0	2008	17.0
	Plant bed 5A<br>**Scenario c)** Increased [CO_2] effects on HoC, lignin + 4°C increase	7.0	2008	19.0
	Plant bed 5A<br>**Scenario d)** Increased [CO_2] effects on HoC, lignin + 11°C increase	7.0	2008	25.0
Phase 3 (plant bed 5B)	**Scenario a)** 'Control'	2.6	332	3.0
	**Scenario b)** Increased [CO_2] effects on HoC only	2.0	202	2.0
	**Scenario b)** Increased [CO_2] effects on HoC + lignin only	2.0	207	2.0
	**Scenario c)** Increased [CO_2] effects on HoC only + 4°C increase	2.0	202	2.0
	**Scenario c)** Increased [CO_2] effects on HoC, lignin + 4°C increase	2.0	207	2.0
	**Scenario d)** Increased [CO_2] effects on HoC only + 11°C increase	2.0	202	3.0
	**Scenario d)** Increased [CO_2] effects on HoC, lignin + 11°C increase	2.0	207	3.0

Table S5 – Results from BEHAVEPLUS model runs adjusted for varying vegetation type, understory and leaf morphology (as described in methods), using an adjusted mid-flame windspeed of 11.2 km/h.
As previously, plant beds 1, 1.5, 2, 3, 4 and 5A model run using a broad-leaved fuel model, that includes an understory TU5, where TU is an Timber Understory fuel model (see Scott and Burgan, 2005). Plant bed 5B run using narrow, needle leaf fuel model TL8, where TL is a timber litter fuel model (See Scott and Burgan, 2005) to capture change in fuel morphology (Belcher et al., 2010; Belcher, 2016). Plant bed 5A and 5B model runs were conducted under 4 scenarios: a. 'Control' conditions with no [CO_2] or temperature change effects, only fuel changes; b. increased [CO_2] effects on Heat of Combustion (HoC) only; c. increased [CO_2] effects on HoC and a 4°C global temperature rise, and d. increased [CO_2] effects on HoC and an 11°C local summer temperature rise.

References

Ainsworth A., Kauffman J.B., Litton C.M. 2010. Interactions between fire and non-native species in Hawaiian forests and shrublands, *Hawai'i Forest Journal* **5**,1-3.

Belcher C.M. 2016. The influence of leaf morphology on litter flammability and its utility for interpreting palaeofire, *Philosophical Transactions B, Royal Society* **371**, 20150163.

Belcher C.M., Hudspith V.A. 2017. Changes to Cretaceous surface fire behaviour influenced the spread of the early angiosperms, *New Phytologist* **213**, 1521-1532.

Belcher C.M. Mander L., Rein G., Jerviz F.X., Haworth M., Hesselbo S.P., Glasspool I.J., McElwain J.C. 2010. Increased fire activity at the Triassic/Jurassic boundary in Greenland due to climate-driven floral change, *Nature Geoscience* **3**, No. 6, 1-4.

Huynh T.T., Poulsen C.J. 2005. Rising atmospheric CO_2 as a possible trigger for the end-Triassic mass extinction, *Palaeogeography, Palaeoclimatology, Palaeoecology* **217**, 223-242

Lenton T.M. Daines S.J., Mills B.J.W. 2018. COPSE reloaded: An improved model of biogeochemical cycling over Phanerozoic time, *Earth-Science Reviews* **178**, 1-28.

McElwain J.C., Beerling D.J., Woodward F.I. 1999. Fossil Plants and Global Warming at the Triassic-Jurassic Boundary. *Science* **285**. 1386-1390.

McElwain J.C., Popa M.E., Hesselbo S.P., Haworth M., Surlyk F. 2007. Macroecological responses of terrestrial vegetation to climatic and atmospheric change across the Triassic/Jurassic boundary in East Greenland. *Paleobiology* **33(4)**, 547-573.

Scott J.H., Burgan R.E. 2005. Standard Fire Behaviour Fuel Models: A Comprehensive Set for Use with Rothermel's Surface Fire Spread Model, *General Technical Report RMRS-GTR-153, United States Department of Agriculture, Forest Service, Rocky Mountain Research Station.*

Simpson K.J., Ripley B.S., Christin P-A., Belcher C.M., Lehmann C.E.R., Thomas G.H., Osborne C.P. 2016. Determinants of flammability in savanna grass species, Journal of Ecology, 104, 138-148, doi: 10.1111/1365-2745.12503

Watson A.J., Lovelock J. 2013. *Fire Phenomena and the Earth System* (ed. Belcher, C. M.) Wiley-Blackwell Press, West Sussex. 288-308
